# Biological synthesis of fluorescent nanoparticles by cadmium and tellurite resistant Antarctic bacteria: exploring novel natural nanofactories

**DOI:** 10.1186/s12934-016-0477-8

**Published:** 2016-05-06

**Authors:** D. O. Plaza, C. Gallardo, Y. D. Straub, D. Bravo, J. M. Pérez-Donoso

**Affiliations:** BioNanotechnology and Microbiology Laboratory, Center for Bioinformatics and Integrative Biology (CBIB), Facultad de Ciencias Biológicas, Universidad Andres Bello, República # 239, Santiago, Chile; Facultad de Ciencias Químicas y Farmacéuticas, Universidad de Chile, Sergio Livingstone Pohlhammer # 1007, Santiago, Chile; Laboratorio de Microbiología Oral, Facultad de Odontología, Universidad de Chile, Sergio Livingstone Pohlhammer # 943, Santiago, Chile

**Keywords:** Fluorescent nanoparticles, Quantum dots, Green synthesis, Antarctica, Bacteria, Heavy metals

## Abstract

**Background:**

Fluorescent nanoparticles or quantum dots (QDs) have been intensely studied for basic and applied research due to their unique size-dependent properties. There is an increasing interest in developing ecofriendly methods to synthesize these nanoparticles since they improve biocompatibility and avoid the generation of toxic byproducts. The use of biological systems, particularly prokaryotes, has emerged as a promising alternative. Recent studies indicate that QDs biosynthesis is related to factors such as cellular redox status and antioxidant defenses. Based on this, the mixture of extreme conditions of Antarctica would allow the development of natural QDs producing bacteria.

**Results:**

In this study we isolated and characterized cadmium and tellurite resistant Antarctic bacteria capable of synthesizing CdS and CdTe QDs when exposed to these oxidizing heavy metals. A time dependent change in fluorescence emission color, moving from green to red, was determined on bacterial cells exposed to metals. Biosynthesis was observed in cells grown at different temperatures and high metal concentrations. Electron microscopy analysis of treated cells revealed nanometric electron-dense elements and structures resembling membrane vesicles mostly associated to periplasmic space. Purified biosynthesized QDs displayed broad absorption and emission spectra characteristic of biogenic Cd nanoparticles.

**Conclusions:**

Our work presents a novel and simple biological approach to produce QDs at room temperature by using heavy metal resistant Antarctic bacteria, highlighting the unique properties of these microorganisms as potent natural producers of nano-scale materials and promising candidates for bioremediation purposes.

## Background

Recently, the vastly explored field of nanotechnology has become a key area of technological development [1]. In the last decade, fluorescent colloidal nanoparticles or quantum dots (QDs) have been intensively studied for both basic and applied research due to their unique size-dependent properties [2]. These nanoparticles are crystalline arrangements sized 1–20 nm, composed by elements of groups II–IV, III–V or IV–VI (such as Cd, Te, Se, Zn, In, As), which form a core covered by an external layer or envelope [3]. In comparison to organic fluorophores, QDs are brighter, more photostable, and display a narrower fluorescence emission spectra allowing multiplexing applications upon excitation by a single wavelength [4].

Currently, QDs are used on several biological, biomedical, optical and optoelectronic applications, ranging from biosensors to solar cells [5, 6]. QDs synthesis is one of the key challenges for applications development, since it determines the size, shape, and surface properties of nanoparticles [7]. Traditionally, the synthesis has been performed using both physical and chemical approaches [8], which are often expensive and harmful to the environment due to the use of high temperatures, inert atmospheres, and toxic reagents [9]. The development of greener (environmentally friendly) synthesis methods has been proposed as an alternative to solve these drawbacks, since they involve low costs, the absence of toxic chemicals, high performance, and produce nanomaterials with well-defined sizes and shapes [10].

The use of biological systems has emerged as a promising alternative to produce water-soluble nanoparticles because they are economic, eco-friendly, easily scaled up, free of toxic compounds, biocompatible and the synthesis is carried out under room conditions [11]. A wide range of organisms, including plants, worms, fungi, yeasts, and bacteria can synthesize QDs from different metal compounds [12, 13]. Prokaryotes posses outstanding additional advantages for QDs synthesis over eukaryotic systems such as faster growth rates and efficient strategies to overcome the inherent toxicity of heavy metals that constitute the core of these nanoparticles, which are very harmful to many organisms at low concentrations [14, 15]. These strategies likely include processes such as metal reduction and/or precipitation, generating nontoxic or less toxic metal nanoarrays [16, 17].

Glutathione (GSH) is a fundamental biological thiol involved in cell protection against different stresses, for example the stress produced by harmful metals [18]. This thiol is depleted by oxidative stress generators such as cadmium and tellurite ions [19, 20], which are usually used as substrates in QDs biosynthesis. A positive relationship between GSH content and the synthesis of nanocrystals has been reported in microbial QDs production [21, 22], highlighting the essential role of cellular antioxidant defenses for QDs generation.

Antarctica has recently emerged as an extraordinary source of microorganisms with exceptional antioxidant defenses. This challenging continent, considered the coldest and driest place on the planet, also presents other extreme conditions such as high levels of solar ultraviolet radiation, low nutrient availability, high salinity and long darkness periods, which increase the generation of oxidative stress. This unique mixture of stressful conditions limits the development of biological communities [23]. Despite this severe scenario, the presence of microorganisms is abundant [24, 25]. These organisms display specialized defense mechanisms against reactive oxygen species (ROS) that maintain their redox state under constant environmental stress [26, 27]. Some enzymatic and non enzymatic adaptations described on Antarctic bacteria are: glutathione S-transferase [28], glutathione reductase [29], thioredoxin [30], catalases [31], superoxide dismutases [32], carotenoids [33] and oxide reductases [34]. Furthermore, the special abilities of some Antarctic bacteria to produce different nanostructures have been recently reported [29, 35, 36]. Thus, the extreme conditions of Antarctica would allow the development of bacteria with unparalleled capacity to cope with high concentrations of heavy metals and synthesize QDs naturally.

Herein, we report cadmium and tellurite resistant bacteria isolated from Antarctica with the ability to produce QDs at room temperature by using high concentrations of these oxidizing substrates. Different bacterial strains showed inherent capabilities for synthesizing fluorescent nanostructures with no additional reagents, representing a promising environmentally friendly methodology to produce valuable biological nanoparticles.

## Results

### Isolation and selection of cadmium and tellurite resistant bacteria

**Fig. 1:**
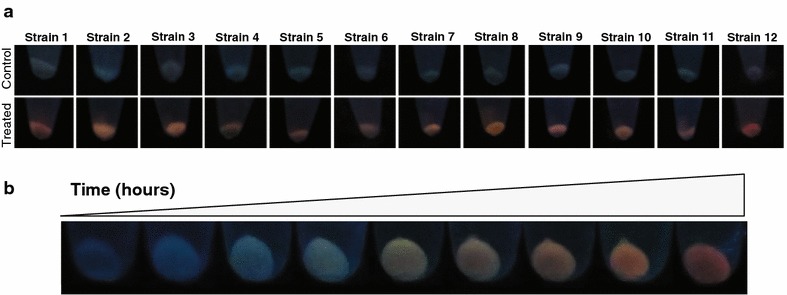
Fluorescence of bacterial pellets under QDs biosynthesis conditions. **a** Cell pellets of the 12 resistant bacterial isolates producing QDs after 48 h treatment. **b** *Fluorescence color* of cell pellets from one representative bacterial isolate (strain 3) across time. Cells were treated with CdCl_2_ (10 mg/L) in PBS buffer and pellets were exposed to UV light (365 nm) to detect fluorescence emission

A total of 410 bacterial isolates were recovered from Antarctic samples using different culture media (see methods). Most of the isolates (92.3 %) were able to grow in LB medium, being selected for further assays. After, aiming to find bacteria for QDs biosynthesis at room temperature, those isolates able to grow at 15–28 °C (217) were chosen for assessing their metal resistance. Among these 217 isolates, a total of 35 resistant to CdCl_2_ (200 mg/L), 44 to K_2_TeO_3_ (50 mg/L) and 16 to both toxic salts were obtained. The 16 isolates were then subjected to an initial biosynthesis test (exposure to CdCl_2_ as previously described by Gallardo et al. [36]), to work only with QDs producing isolates. Finally, 12 resistant isolates displayed fluorescence within 24–48 h after cadmium exposure (Fig. 1a). Time dependent changes on the fluorescence were evaluated on one representative isolate (Fig. 1b). Changes in color emission across the incubation time (moving from green to red) were observed in bacterial pellets excited at 360 nm. This behavior is a key feature of QDs as has been reported previously [36]. Thus, these 12 isolates were selected for subsequent experiments.

### Identification of metal-resistant bacteria

**Fig. 2:**
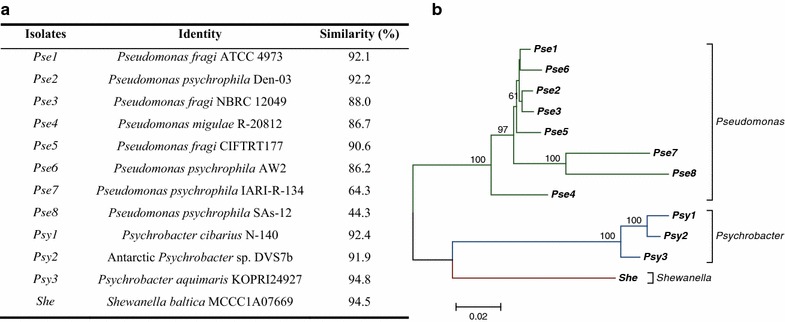
Taxonomic identification of Antarctic resistant bacteria based on 16S rRNA gene sequences. **a** Assignation of most closely related reference strains to each resistant isolate using the Ribosomal Data Project (RDP) database. **b** Neighbor-joining tree showing the relationships among the 12 resistant bacteria. *Numbers* above branches indicate bootstrap resampling coefficients (>50 %) from 1000 replications. *Scale bar* corresponds to 0.02 substitutions per nucleotide position

The results of 16S rRNA gene sequence analysis of the 12 metal-resistant bacteria revealed that they belong to genera *Pseudomonas* (eight isolates), *Psychrobacter* (three isolates) and *Shewanella* (one isolate), being renamed as *Pse*, *Psy*, and *She*, respectively. Some isolates evidenced low similarity scores to known species (*Pse7* and *Pse8*), representing probably species not yet described (Fig. 2a). Phylogenetic analysis showed three clear clustering patterns in terms of genera and evidenced remarkable genetic distances among selected isolates (Fig. 2b).

### Growth and metabolic characteristics of metal-resistant bacteria

Phenotypic characterization of the 12 resistant bacterial isolates evidenced differences among them (Table 1). The optimal growth temperature for the 12 isolates was 28 °C, nonetheless, isolates were able to grow at temperatures ranging from 10–28 °C. No growth was observed at 37 °C. All isolates were Gram-negative, mostly with homogeneous cream-colored colonies surrounded by abundant mucus. All selected isolates were resistant to Lincomycin and sensitive to Gentamycin and Kanamycin.

**Table 1:** Characterization and growth conditions of metal-resistant bacteria

	Strains											
	*Pse1*	*Pse2*	*Pse3*	*Pse4*	*Pse5*	*Pse6*	*Pse7*	*Pse8*	*Psy1*	*Psy2*	*Psy3*	*She*
Optimal T (°C)	28	28	28	28	28	28	28	28	28	28	28	28
Gram staining	−	−	−	−	−	−	−	−	−	−	−	−
Cell shape	R	R	R	R	R	R	R	R	Cb	Cb	Cb	R
Colony color	c	c	c	c	c	c	c	c	c	c	c	r
Ampicillin	+	+	+	+	+	+	+	+	−	−	−	−
Colistin	−	−	−	−	−	−	−	−	−	−	−	+
Fosfomycin	+	+	+	+	+	+	+	+	+	+	+	+
Gentamycin	−	−	−	−	−	−	−	−	−	−	−	−
Kanamycin	−	−	−	−	−	−	−	−	−	−	−	−
Lincomycin	+	+	+	+	+	+	+	+	+	+	+	+
Oxolinic acid	+	+	−	−	−	+	−	−	−	−	−	−
Polymixin B	−	−	−	−	−	−	−	−	−	−	−	+
Rifampicin	+	+	+	+	+	+	+	+	−	−	−	(+)
Sulfonamide	+	+	+	+	+	+	+	+	−	+	+	+

*T* temperature; *R* rod-shaped, *Cb* coccobacilli; *c* cream, *r* reddish

Antibiotic sensitivity: resistant strain = +, sensitive strain = − and intermediate = (+)

The metabolic profile reveals a high diversity among all selected isolates, particularly in terms of enzymatic activities, excepting β-galactosidase (negative for all isolates; Table 2). The predominant activities were nitrate reductase and indole production (tryptophanase). In terms of substrate utilization, a transversal use of substrates was determined. All isolates were able to metabolize glucose, arabinose, gluconate, and malic acid.

**Table 2:** Metabolic profiles of metal-resistant bacteria

	Strains											
	*Pse1*	*Pse2*	*Pse3*	*Pse4*	*Pse5*	*Pse6*	*Pse7*	*Pse8*	*Psy1*	*Psy2*	*Psy3*	*She*
NO_3_ reductase	+	+	−	+	−	+	−	+	+	+	+	+
Tryptophanase	+	+	−	+	−	+	−	+	−	−	+	+
Glu fermentation	−	−	+	+	+	−	+	−	+	−	−	−
Arg dihydrolase	+	+	+	−	+	+	+	+	−	−	−	−
Urease	+	+	+	−	+	+	+	+	−	−	−	−
Gelatinase	−	−	−	+	+	−	−	−	−	−	−	+
β-glucosidase	−	−	+	−	−	−	−	−	−	−	−	+
β-galatosydase	−	−	−	−	−	−	−	−	−	−	−	−
d-Glucose	+	+	+	+	+	+	+	+	+	+	+	+
D-Manose	+	+	+	+	+	+	+	+	+	+	+	−
D-Manitol	+	+	+	+	+	+	+	+	+	+	+	−
d-Maltose	−	−	−	+	+	−	+	−	+	+	+	+
L-Arabinose	+	+	+	+	+	+	+	+	+	+	+	+
NAG	+	+	−	+	+	+	+	−	+	+	+	+
Gluconate	+	+	+	+	+	+	+	+	+	+	+	+
Capric acid	+	+	+	+	+	+	+	+	+	−	−	−
Adipic acid	−	−	−	+	+	−	+	−	+	+	+	−
Malic acid	+	+	+	+	+	+	+	+	+	+	+	+
Phenylacetic acid	−	−	−	+	+	−	+	−	+	−	−	+
Citrate trisodium	+	+	−	+	+	+	+	+	+	−	−	+

*Glu* Glucose; *Arg* Arginine; *NAG* N-acetylglucosamine

### Metal resistance levels of selected bacteria

MIC values for the 12 resistant bacteria ranged between 500–1400 and 62.5–1200 mg/L for CdCl_2_ and K_2_TeO_3_, respectively (Table 3). Cadmium and tellurite resistant bacteria presented elevated MIC values, which in some cases exceed seven and twenty times the concentrations used initially for selection, respectively. The strains that showed the highest cadmium resistance level were *Pse1*, *Pse3* and the three *Psychrobacter* spp. (1400 mg/L), whereas for tellurite were *Pse3* and *Psy1* (1200 mg/L).

**Table 3:** Cadmium and tellurite MICs of metal-resistant Antarctic bacteria

	Strains											
	*Pse1*	*Pse2*	*Pse3*	*Pse4*	*Pse5*	*Pse6*	*Pse7*	*Pse8*	*Psy1*	*Psy2*	*Psy3*	*She*
CdCl_2_	1400	500	1400	500	500	1000	1000	1000	1400	1400	1400	500
K_2_TeO_3_	400	125	1200	62.5	400	125	400	600	1200	500	125	125

Concentrations are expressed as mg/L

### Biosynthesis of fluorescent nanoparticles

**Fig. 3:**
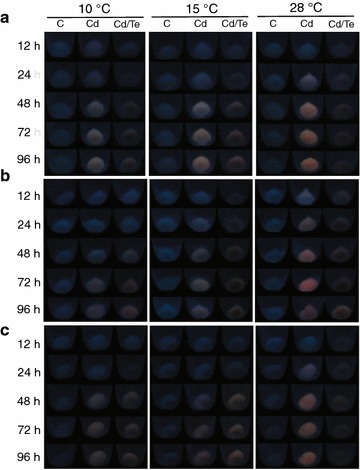
Effect of different incubation temperatures on QDs biosynthesis. The fluorescence of bacterial cells exposed to metals at different temperatures was evaluated. **a** *Pse3*, **b** *Pse8* and **c** *Psy1*. C = control (untreated cells), Cd = cells treated with CdCl_2_ (10 mg/L) and Cd/Te = cells treated with CdCl_2_ (10 mg/L) + K_2_TeO_3_ (0.5 mg/L). Cell pellets were exposed to UV light (365 nm) to detect the fluorescence of produced QDs across time

Based on MICs and the fluorescence determined in Fig. 1, we selected *Pse3*, *Pse8* and *Psy1* isolates to assess their capacity to biosynthesize QDs under different temperatures and metal concentrations. Optimal conditions for nanoparticles bioproduction such as incubation temperature and metal concentrations were determined. The experiments were carried out by two treatments: exposing bacteria to (a) CdCl_2_ (10 mg/L) or (b) CdCl_2_ (10 mg/L) + K_2_TeO_3_ (0.5 mg/L). The effect of incubation temperature was investigated at 10, 15 and 28 °C during 96 h (Fig. 3). A QDs-characteristic change on fluorescence emission color, moving from green to red, was observed in bacterial pellets over time, with slight differences among strains. The fluorescence of cells exposed to CdCl_2_ increased with temperature, evidencing an optimal biosynthesis temperature of 28 °C for the three bacterial strains evaluated. Low fluorescence was observed in cells treated with CdCl_2_ + K_2_TeO_3_, particularly when compared to CdCl_2_ exposed cells. In addition, a black precipitate was observed, most probably corresponding to Teº generated by Te^4+^ reduction [37].

**Fig. 4:**
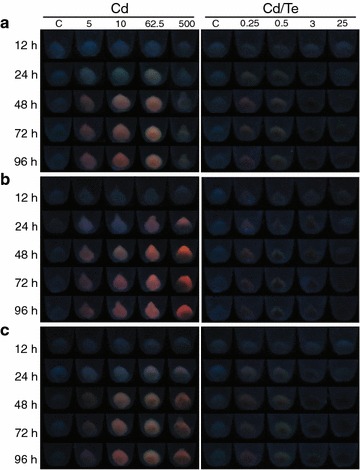
Effect of different heavy metal concentrations on QDs biosynthesis. Bacterial cell pellets of selected Antarctic strains were exposed to UV light (365 nm) to detect the fluorescence of produced QDs over time. **a** *Pse3*, **b** *Pse8* and **c** *Psy1*. *Treatments* C = control (untreated) cells, Cd = cells treated with CdCl_2_ (5–500 mg/L) and Cd/Te = cells treated with CdCl_2_ (10 mg/L) + K_2_TeO_3_ (0.25–25 mg/L)

Selected bacteria were then exposed to high concentrations of CdCl_2_ and K_2_TeO_3_. The concentrations of both metals assayed for QDs biosynthesis were chosen based on MICs and bacterial growth curves of each metal-resistant isolate (data not shown). Four concentrations of CdCl_2_ (5, 10, 62.5 and 500 mg/L) and K_2_TeO_3_ (0.25, 0.5, 3, and 25 mg/L) were evaluated and bacterial cultures were incubated at 28 °C for 4 days (Fig. 4).

In agreement with results of Fig. 3, strains treated only with CdCl_2_ presented higher fluorescence than those exposed to both salts. The *Pse3* strain exposed to CdCl_2_ presented fluorescence at three concentrations (5, 10, and 62.5 mg/L) (Fig. 4a), whereas *Pse1* and *Psy1* strains emitted fluorescence at all CdCl_2_ concentrations tested, including 500 mg/L (Fig. 4b, c). For the treatment with both salts, at any K_2_TeO_3_ concentration evaluated the fluorescence emitted from the three strains was strongly affected, and increased tellurite concentrations were positively correlated with increasing black precipitates (black spots in the pellets).

### Transmission electron microscopy

**Fig. 5:**
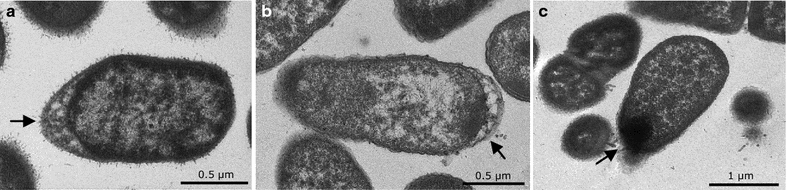
Transmission electron microscopy of cells biosynthesyzing QDs. Ultrathin sections of Antarctic bacteria during QDs biosynthesis (cells were exposed to 50 mg/L CdCl_2_ for 36 h). **a** *Pse3*, **b** *Pse8,* and **c** *Psy1*. *Arrows* indicate bacterial endings where electron-dense nanostructures and vesicles were observed

To study the effects of biosynthetic conditions on cellular ultrastructure of the three resistant bacteria, transmission electron microscopy (TEM) was performed on cells exposed to CdCl_2_ (condition in which the highest fluorescence emission was obtained). The micrographs obtained from TEM evidenced different changes in cell morphology and ultrastructure of the three strains tested (Fig. 5). The major changes were observed in both, cell envelope and poles. In *Pseudomonas* strains exposed to cadmium (*Pse3* and *Pse1*), an increase of periplasmic space, particularly at the cell poles, was observed (arrows of Fig. 5a, b). Moreover, a high number of structures resembling outer membrane vesicles (OMV) at the cell periphery were detected. In addition, the presence of electron-dense nanostructures was observed at the bacterial endings, particularly in the periplasmic space of *Pse1* (Fig. 5b, arrows).

### Spectroscopic characterization of biosynthesized nanoparticles

**Fig. 6:**
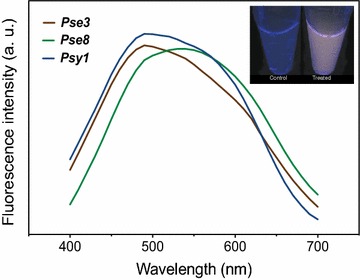
Fluorescence spectra of purified QDs. Fluorescence emission spectra of biosynthesized nanoparticles produced by selected Antarctic bacteria (exc. 365 nm). *Inset* shows a representative QDs purification obtained from strain *Pse3*. *Treatments* C = control (untreated cells), Cd = cells treated with CdCl_2_ (50 mg/L)

The optical properties of QDs produced by the three resistant strains were analyzed by determining the absorption and fluorescence spectra (Fig. 6). The inset of the figure shows the fluorescence of a representative solution after purification. Absorption spectra were similar for QDs produced by all strains displaying increased absorption at 400 nm as reported for biosynthesized CdS nanoparticles (not shown) [21, 36]. Regarding the emission spectra, wide peaks between 450–600 nm when excited at 365 nm were observed. All spectroscopic characteristics of purified QDs correspond to those previously reported for CdS biosynthesized nanoparticles [21, 36, 38, 39].

## Discussion

The selection process of cadmium and tellurite resistant bacteria from Antarctica revealed an extensive number of isolates resistant to each salt. After the initial QDs biosynthesis test, 12 resistant isolates displayed the ability to generate these fluorescent nanoparticles under the conditions evaluated. The 16S rRNA gene analysis classified the 12 resistant isolates up to genus level. All belong to Gammaproteobacteria, a class that predominates in several Antarctic environments [40]. Based on optimal growth temperatures, the 12 selected isolates were classified as psychrotolerant bacteria [41]. Some records have uncovered a predominance of psychrotolerant bacteria over psychrophilic ones in habitats dominated by low temperatures [42].

The extensive mucus secreted by most bacterial colonies, a characteristic fairly extended in Antarctic bacteria [43], could facilitate the process of QDs biosynthesis by trapping heavy metals. This could directly favor the process of nanoparticle nucleation and/or constitute a strategy to deal with the toxics.

Regarding a potential enzymatic activity linked to QDs biosynthesis, just the NADH-dependent enzyme nitrate reductase has been previously associated to a high production of silver nanoparticles in *Bacillus subtilis* [44] and *B. licheniformis* [45], whereas in *Rhodobacter sphaeroides* this enzyme had a central effect over size and shape of the nanoparticles [46]. Likewise, nitrate reductase is implicated in the generation of PbO and Se nanoparticles in *Enterobacter* sp., *B. anthracis* [47] and *Halococcus salifodinae* [48]. Interestingly, not all resistant bacteria displayed nitrate reductase activity, suggesting that other enzymes or molecules may be involved in the QDs biosynthetic process described here.

Regarding heavy metal resistance, the 12 resistant bacteria showed MIC values ranging from 62.5–1200 mg/L. These results indicate the presence of highly resistant bacteria, particularly if compared with resistance levels reported for several eukaryotic and prokaryotic cells (MICs between 1–50 mg/L) [37]. It has been reported that Antarctic bacteria are able to cope and/or adapt to pollutants even in low human-impacted environments [49–51], illustrating their stunning attributes as promising candidates for bioremediation purposes.

In Antarctica, most trace elements have a natural origin, and the accumulation of noxious metals have been subject of extensive controversy. Recently, it has been advertised that an increase in cadmium levels is linked to human activities [52]. Additionally, some penguins possess ways of absorption, elimination and bioaccumulation of this harmful element, which would support its presence in the Antarctic ecosystem [53]. These precedents suggest that Antarctica is not free of moderate cadmium anthropogenic pollution [54]. In the case of tellurite, no reports showing its presence in Antarctic territory have been published. Despite this, Arenas et al. [55] isolated some tellurite-resistant bacteria, emphasizing their remarkable ability to cope with such a highly oxidative toxicant.

The synthesis of QDs can be easily detected by specific color changes during cell incubation, [56]. The intracellular fluorescence color shift observed in all resistant bacteria across time, from green to red, it is a unique feature of these nanocrystals [3, 21, 36].

To date, the mechanisms involved in biological synthesis of QDs remain poorly understood. Chemical methods follow steps involving nucleation and crystals growth [57]. On the other hand, QDs biosynthesis appears to be more complex. In general, bacterial synthesis is achieved by a reduction step followed by metal precipitation [58]. Some studies suggest that some bacterial defense mechanisms against oxidative stress generated by toxic metals, as thiols, could direct the formation of QDs [21, 59]. In this thiol-oxidizing interaction, the oxidant is neutralized by conversion to a less toxic byproduct. During the cell-response to the stress generated by Hg, Ag, As, Pb and Cd, GSH forms spontaneously ion-conjugates, which irreversibly depletes intracellular metal concentration [59, 60]. For the biosynthesis of CdTe nanocrystals, Te^2−^ ions are required to form the nanoparticle core [58]. Nevertheless, in our results, an intracellular black precipitate evidenced a marked reduction of tellurite to Teº, as widely reported in Gram-negative tellurite resistant bacteria [37]. Accordingly, the low fluorescence observed in bacterial cells treated with the mixture of cadmium and tellurite salts, may be due to the formation of CdS nanocrystals instead of CdTe, probably as consequence of the lack of Te^2−^ ions and an accelerated precipitation to Teº. Lately, Monrás et al. [21] pointed out the vital importance of thiols, especially GSH, and the cellular redox state for CdS and CdTe production in *Escherichia coli*. These antecedents support the astonishing antioxidant defenses of resistant bacteria from Antarctic as enhanced QDs producers.

A fundamental parameter for bioproduction of QDs is the temperature. Most CdS nanocrystals microbial synthesis reported to date are carried out at 37 °C [15]. Some studies showing lower synthesis temperatures than 37 °C have used different bacterial strains such as *Lactobacillus* sp. [39], *R. palustris* [61], *R. sphaeroides* [46], *Brevibacterium casei* [62] and some Antarctic strains [36]. Nonetheless, heretofore no bacterial strain has biosynthesized CdS nanocrystals at as high concentrations of CdCl_2_ as determined in the present study, evidencing the enormous potential of Antarctic bacteria like powerful QDs producers and bioremediation agents in simultaneous processes at room temperature. Furthermore, most biosynthesis protocols using different microorganisms and plant extracts use an external source of S, as NaS [63], whereas in the procedure described here just metal precursors are added (no other additional reagent is required). Hence, the fundamental advantages of using Antarctic bacteria over previous bacterial QDs methods rely on avoiding the use of any additional reagents, room conditions to carry out the synthesis process, and producing nanoparticles using higher metal concentrations. We foresee that Antarctic microorganisms will contribute to a new era in future industrial production of nanomaterials and in the implementation of bioremediation tools.

Changes on the cellular ultrastructure were observed for the three representative isolates under QDs biosynthesis conditions, indicating physiological adaptations, essentially at the cell poles, as previously described for silver nanoparticles [16] and QDs [21]. The nanometric structures present in the cells endings subjected to QDs biosynthesis conditions were similar to those previously observed in the biosynthesis of Ag NPs [36]. The presence of structures similar to OMV is also observed. Cells utilize OMV to deal with diverse environmental stresses and their presence in NPs biosynthesis has been reported [43, 64–67]. The formation of OMV in *R. sphaeroides* has been linked to the transport of CdS nanostructures [46]. In this context, the production of OMV could be stimulated during biosynthesis of QDs as a way to eliminate nanoparticles from cells (entrapped within vesicles).

Finally, the wide fluorescence emission spectra of purified nanoparticles might be consequence of a high size-dispersion of nanoparticles [15, 68]. This is a typical behavior of QDs biosynthesis most probably related with differences in the metabolic state of culture cells when interacting with metals. In addition, time depending changes in emission colors (moving from green to red) observed in cell pellets excited at 360 nm is a characteristic feature of biosynthesized QDs [36]. Altogether, size and spectroscopic characteristics displayed on NPs produced by Antarctic bacteria after metal-exposure confirm the generation of fluorescent nanoparticles.

## Conclusions

The present study demonstrates the successful synthesis of fluorescent nanoparticles by highly cadmium and tellurite resistant bacteria isolated from Antarctica. This ecofriendly approach was carried out under room conditions and higher concentrations of heavy metals than those previously reported. Further research will be critical to understand the biochemical and molecular mechanisms underlying the nanoparticles biosynthesis in order to identify key components that could improve the technology.

Finally, our findings provide meaningful insights for the development of greener strategies for QDs production and highlight the unique properties of microorganisms from Antarctica as potent natural producers of nano-scale materials and promising candidates for bioremediation purposes.

## Methods

### Sampling

Samples were collected during the 48th Chilean Antarctic Expedition, sponsored by the Chilean Antarctic Institute (INACH) in January 2012. Environmental samples of seawater, soil, sediments and ice were obtained from King George, Greenwich, Livingston, Deception, and Southern Shetlands Islands. Samples were taken from each site and placed in sterile 50 mL Falcon tubes and cold stored until their use.

### Isolation of bacteria

100 mg (solid) or 100 µL (liquid) of samples were suspended on 1 mL (final volume) of sterile distilled water and stirred by vortexing for 30 min. Then, 10 µL of each suspension was used to inoculate 990 µL of different growth media, including Luria–Bertani (LB, 1X and 0.1X), Saline LB (4 % NaCl), R2A [69], ATTC [70] and marine medium (1 g yeast extract, 10 g tryptone, 5 g peptone in 1 L of sterilized sea water), incubated at 4–37 °C for 24–48 h. Pure bacterial cultures were obtained after successive transfers of single colonies to the corresponding medium and then stored by freezing at −80 °C in liquid medium with 30 % (v/v) glycerol.

### Selection of cadmium and tellurite resistant bacteria

Bacterial isolates were screened by plating on LB agar supplemented with 200 mg/L CdCl_2_ and 50 mg/L K_2_TeO_3_ (Sigma-Aldrich). Bacterial growth was evaluated after 24–48 h, and bacteria able to grow in both culture media were separated from the rest and designed as resistant isolates.

### Optimal growth temperature

Resistant bacterial isolates were grown in LB medium until OD_600_ = 0.2 was reached. Serial dilutions were performed in 96-well microplates and 5 µL of each dilution were used to inoculate LB plates. The incubation temperatures assayed were 4, 10, 15, 28 and 37 °C and bacterial growth was checked after 24 h inoculation.

### Identification of resistant bacteria

Metal-resistant bacterial isolates were taxonomically classified by 16S rRNA gene sequencing analysis. For gene amplification a Colony PCR kit (Bionix Red, BioLine) was used. A single colony of each isolate was heated at 95 °C for 10 min to disrupt the cells. PCR reaction was performed using universal primers U515 F [71] and U1492 R [72] as follows: pre-heating 5 min at 95 °C, followed by 25 cycles of denaturation at 95 °C for 30 s, annealing 30 s at 55 °C and extension 30 s at 72 °C, plus a final extension at 72 °C for 10 min. PCR products were evaluated by agarose gel (1 %) electrophoresis (stained with GelRed) and sequenced by Macrogen (Korea). Presence of chimeric sequences were discarded by DECIPHER tool [73]. The sequences were analyzed using tools from Ribosomal Database Project (RDP) (http://www.rdp.cme.msu.edu) [74].

Sequences were aligned using MUSCLE method [75] and grouped according to their closest neighbors in phylogenetic trees by neighbor-joining method. Distances were determined using the Maximum Likelihood by software MEGA v.6.0.6 [76]. The reproduction of each branch was performed by 1000 bootstrap analyses.

### Growth and metabolic characteristics of resistant bacteria

Individual characteristics of selected bacterial colonies were evaluated using LB medium at 28 °C. Cell characterization was performed by Gram staining and by observation of morphology using a light microscope. Growth of resistant bacteria under differents antibiotics was tested by Kirby-Bauer method [77] using 10 comercial strips (Valtek) for 24 h.

Metabolic characterization consisted in assaying substrates utilization and acid production from various sugars using API 20NE strips (BioMérieux Inc. Durham, NC). The results were obtained after 24–48 h inoculation. All assays were carried out according to the manufacturer indications.

### Minimal inhibitory concentration

MIC values for cadmium and tellurite were determined by the method previously described by Pérez et al. [37]. Briefly, 10,000 mg/L of CdCl_2_ and K_2_TeO_3_ stock solutions were placed in 300 µL of LB medium. Then, serial dilutions were performed in 96-well microplates and inoculated with 5 µL of bacterial cultures grown previously until an OD_600_ = 0.5. Microplates were incubated at 28 °C and bacterial growth was evaluated after 24–48 h.

### Biosynthesis of fluorescent nanoparticles

QDs biosynthesis was carried out following the protocol reported by Monrás et al. [21]. Briefly, resistant bacteria were grown overnight in LB medium at 28 °C until an OD_600_ ~0.5 was reached. Then, cells were collected and resuspended in two vials with phosphate-buffer saline (PBS) 50 mM pH 7.4, one containing CdCl_2_ (10 mg/L) and other CdCl_2_ (10 mg/L) + K_2_TeO_3_ (0.5 mg/L). Both treatments were incubated at 28 °C by 5 days. Each 12 h tubes were centrifuged at 12,000 rpm for 5 min, and QDs production (fluorescence of cellular pellets) was evaluated by UV light (365 nm) exposition using a transiluminator.

### Transmission electron microscopy

Two strains were selected and treated with CdCl_2_ (10 g/mL; as detailed above), and grown under QDs biosynthesis conditions for 36 h. Fluorescent cells were collected by centrifugation (10,000 rpm for 5 min), fixed using glutaraldehyde 2.5 % in cacodilate buffer 0.1 M pH 7.2 during 6 h at room temperature and washed with the same buffer for 18 h at 4 °C. Samples were fixed with aqueous osmium tetroxide (1 %) and uranile acetate (1 %). Finally, samples were dehydrated and infiltrated with an epoxy resin overnight. Ultrathin Sects. (60–70 nm thick) were obtained using an ultramicrotome (Sorval MT-5000) and placed in copper grids (Formvar carbon 300 mesh, grid hole size of 63 μm). Electron microscopy images were collected using a Phillips Tecnai 12 BioTwin microscope at 80 kV.

### Purification of biosynthesized nanoparticles

Cell disruption was performed by adding 1 N sodium hydroxide (NaOH) to fluorescent bacterial pellets and incubating at 90 °C for 5 min, allowing QDs to stay in solution. Then, tubes were centrifuged at 10,000 g for 10 min in order to discard cellular debris. Aqueous phase (with the nanoparticles) was obtained after a separation of fluorescent fraction with chloroform (2:1) at room temperature. The resultant fraction was further purified by chromatography using a Sephadex G-75 column equilibrated and eluted with PBS buffer 50 mM. All obtained fractions were exposed to UV light (365 nm) to evaluate fluorescence, and then were concentrated using Amicon (10 kDa) tubes by centrifugation at 7000 rpm for 30 min.

### Spectroscopic characterization of biosynthesized nanoparticles

Absorbance and fluorescence spectra of purified nanoparticles were determined by using a multiplate reader Synergy H1 M (Biotek) at room temperature. Emission spectra were obtained after excitation at 365 nm and recorded in the range of 400–700 nm.

## References

[1] Adams FC, Barbante C (2013). Nanoscience, nanotechnology and spectrometry. Spectrochim Acta Part B.

[2] Kovalenko MV, Manna L, Cabot A, Hens Z, Talapin DV, Kagan CR, Klimov XVI, Rogach AL, Reiss P, Milliron DJ, Guyot-Sionnnest P, Konstantatos G, Parak WJ, Hyeon T, Korgel BA, Murray CB, Heiss W (2015). Prospects of nanoscience with nanocrystals. ACS Nano.

[3] Kershaw SV, Susha AS, Rogach AL (2013). Narrow bandgap colloidal metal chalcogenide quantum dots: synthetic methods, heterostructures, assemblies, electronic and infrared optical properties. Chem Soc Rev.

[4] Liu T, Liu B, Zhang H, Wang Y (2005). The fluorescence bioassay platforms on quantum dots nanoparticles. J Fluoresc.

[5] Selinsky RS, Ding Q, Faber MS, Wright JC, Jin S, Sarah R (2013). Quantum dot nanoscale heterostructures for solar energy conversion. Chem Soc Rev.

[6] He X, Ma N (2014). An overview of recent advances in quantum dots for biomedical applications. Colloids Surf B Biointerfaces.

[7] Hines DA, Kamat PV (2013). Quantum dot surface chemistry: ligand effects and electron transfer reactions. J Phys Chem C.

[8] Biju V, Itoh T, Anas A, Sujith A, Ishikawa M (2008). Semiconductor quantum dots and metal nanoparticles: syntheses, optical properties, and biological applications. Anal Bioanal Chem.

[9] Karakoti AS, Shukla R, Shanker R, Singh S (2014). Surface functionalization of quantum dots for biological applications. Adv Colloid Interface Sci.

[10] Kharissova OV, Dias HV, Kharisov BI, Olvera B, Jiménez VM (2013). The greener synthesis of nanoparticles. Trends Biotechnol.

[11] Mohanpuria P, Rana NK (2008). Biosynthesis of nanoparticles: technological concepts and future applications. J Nanopart Res.

[12] Thakkar KN, Mhatre SS, Parikh RY (2010). Biological synthesis of metallic nanoparticles. Nanomedicine.

[13] Durán N, Marcato PD, Durán M (2011). Mechanistic aspects in the biogenic synthesis of extracellular metal nanoparticles by peptides, bacteria, fungi, and plants. Appl Microbiol Biotechnol.

[14] Sweeney RY, Mao C, Gao X, Burt JL, Belcher AM, Georgiou G, Iverson BL (2004). Bacterial biosynthesis of cadmium sulfide nanocrystals. Chem Biol.

[15] Jacob JM, Lens PNL, Mohan R (2015). Microbial synthesis of chalcogenide semiconductor nanoparticles: a review. Microb Biotechnol.

[16] Klaus T, Joerger R, Olsson E (1999). Silver-based crystalline nanoparticles, microbially fabricated. PNAS.

[17] Narayanan KB, Sakthivel N (2010). Biological synthesis of metal nanoparticles by microbes. Adv Colloid Interface Sci.

[18] Potter AJ, Trappetti C, Paton JC (2012). *Streptococcus pneumoniae* uses glutathione to defend against oxidative stress and metal ion toxicity. J Bacteriol.

[19] Turner RJ, Weiner JH, Taylor DE (1999). Tellurite-mediated thiol oxidation in *Escherichia coli*. Microbiol.

[20] Helbig K, Grosse C, Nies DH (2008). Cadmium toxicity in glutathione mutants of *Escherichia coli*. J Bacteriol.

[21] Monrás JP, Díaz V, Bravo D, Montes RA, Chasteen TG, Osorio-Román IO, Vásquez CC, Pérez-Donoso JM (2012). Enhanced glutathione content allows the in vivo synthesis of fluorescent CdTe nanoparticles by *Escherichia coli*. PLoS One.

[22] Li Y, Cui R, Zhang P, Chen B, Tian Z, Li L, Hu B, Pang D, Xie Z, Chemistry A, Sciences M, Sciences L (2013). Mechanism-oriented controllability of intracellular quantum dots formation: the role of glutathione. ACS Nano.

[23] Chown SL, Clarke A, Fraser CI, Cary SC, Moon KL, Mcgeoch MA (2015). The changing form of Antarctic biodiversity. Nature.

[24] Chong CW, Annie GY, Richard T, Riddle MJ, Tan IKP (2009). DGGE fingerprinting of bacteria in soils from eight ecologically different sites around Casey Station, Antarctica. Polar Biol.

[25] Cowan DA, Makhalanyane TP, Dennis PG, Hopkins DW (2014). Microbial ecology and biogeochemistry of continental Antarctic soils. Front Microbiol.

[26] Chattopadhyay MK, Raghu G, Sharma YVRK, Biju AR, Rajasekharan MV, Shivaji S (2011). Increase in oxidative stress at low temperature in an Antarctic bacterium. Curr Microbiol.

[27] Kulkarni HM, Jagannadham MV (2014). Molecular characterization and functional analysis of outer membrane vesicles from the antarctic bacterium *Pseudomonas syringae* suggest a possible response to environmental conditions. J Proteome Res.

[28] Shi Y, Wang Q, Hou Y, Hong Y, Han X, Yi J, Qu J, Lu Y (2014). Molecular cloning, expression and enzymatic characterization of glutathione S-transferase from Antarctic sea-ice bacteria. Microbiol Res.

[29] Pugin B, Cornejo FA, Muñoz-Díaz P, Muñoz-Villagrán CM, Vargas-Pérez JI, Arenas FA, Vásquez CC (2014). Nanostructures exhibiting antibacterial properties. Appl Environ Microbiol.

[30] Falasca P, Evangelista G, Cotugno R, Marco S (2012). Properties of the endogenous components of the thioredoxin system in the psychrophilic eubacterium *Pseudoalteromonas haloplanktis* TAC 125. Extremophiles.

[31] Tribelli PM, Iustman LJR, Mariela V, Di Martino C, Revale S, Beatriz S, López NI (2012). Genome sequence of the polyhydroxybutyrate producer *Pseudomonas extremaustralis*, a highly stress-resistant Antarctic bacterium. J Bacteriol.

[32] Zheng Z, Jiang Y, Miao J, Wang Q, Zhang B, Li G (2006). Purification and characterization of a cold-active iron superoxide dismutase from a psychrophilic bacterium, *Marinomonas* sp. NJ522. Biotechnol Lett.

[33] Dieser M, Greenwood M, Foreman CM (2010). Carotenoid pigmentation in antarctic heterotrophic bacteria as a strategy to withstand environmental stresses. Arct Antarct Alp Res.

[34] Madonna S, Papa R, Birolo L, Aurore F, Doti N, Marino G, Quemeneur E, Sannia G, Tutino ML, Duilio A (2006). The thiol-disulfide oxidoreductase system in the cold-adapted bacterium *Pseudoalteromonas haloplanktis* TAC 125: discovery of a novel disulfide oxidoreductase enzyme. Extremophiles.

[35] Correa-Llantén DN, Muñoz-Ibacache SA, Castro ME, Muñoz PA, Blamey JM (2013). Gold nanoparticles synthesized by *Geobacillus* sp. strain ID17 a thermophilic bacterium isolated from Deception Island, Antarctica. Microb Cell Fact.

[36] Gallardo C, Monrás JP, Plaza DO, Collao B, Saona LA, Venegas FA, Soto C, Ulloa G, Vásquez CC, Bravo D (2014). Low-temperature biosynthesis of fluorescent semiconductor nanoparticles (CdS) by oxidative stress resistant Antarctic bacteria. J Biotechnol.

[37] Pérez JM, Calderón I, Arenas FA, Fuentes DE, Pradenas GA, Fuentes EL, Sandoval JM, Castro ME, Elías A, Vásquez CC (2007). Bacterial toxicity of potassium tellurite: unveiling an ancient enigma. PLoS One.

[38] Dameron CT, Reese RN, Mehra RK (1989). Biosynthesis of cadmium sulphide quantum semiconductor crystallites. Nature.

[39] Prasad K, Jha AK (2010). Biosynthesis of CdS nanoparticles: an improved green and rapid procedure. J Colloid Interface Sci.

[40] Yu Y, Li H, Zeng Y, Chen B (2010). Phylogenetic diversity of culturable bacteria from Antarctic sandy intertidal sediments. Polar Biol.

[41] De Maayer P, Anderson D, Cary C, Cowan DA (2014). Some like it cold: understanding the survival strategies of psychrophiles. EMBO Rep.

[42] Shivaji S, Begum Z, Soma S, Nageswara S, Thamban M, Krishnan KP, Singh SM, Srinivas TNR (2013). Antarctic ice core samples: culturable bacterial diversity. Res Microbiol.

[43] Frias A, Manresa A, De Oliveira E, López-iglesias C, Mercade E (2010). Membrane vesicles: a common feature in the extracellular matter of cold-adapted Antarctic bacteria. Microb Ecol.

[44] Saifuddin N, Wong CW, Yasumira AAN (2009). Rapid biosynthesis of silver nanoparticles using culture supernatant of bacteria with microwave irradiation. J Chem.

[45] Kalimuthu K, Babu RS, Venkataraman D, Bilal M, Gurunathan S (2008). Biosynthesis of silver nanocrystals by *Bacillus licheniformis*. Colloid Surf B.

[46] Bai H, Yang B, Chai C, Yang G, Jia W, Yi Z (2011). Green synthesis of silver nanoparticles using *Rhodobacter Sphaeroides*. World J Microbiol Biotechnol.

[47] El-Shanshoury AER, Elsilk SE, Ateya PS, Ebeid EM (2012). Synthesis of lead nanoparticles by *Enterobacter* sp. and avirulent *Bacillus anthracis* PS2010. Ann Microbiol.

[48] Srivastava P, Braganca JM, Kowshik M (2014). In vivo synthesis of selenium nanoparticles by *Halococcus salifodinae* BK18 and their anti-proliferative properties against HeLa cell line. Biotechnol Prog.

[49] Lo Giudice A, Casella P, Bruni V, Michaud L (2013). Response of bacterial isolates from Antarctic shallow sediments towards heavy metals, antibiotics and polychlorinated biphenyls. Ecotoxicology.

[50] Lorenz N, Hintemann T, Kramarewa T, Katayama A, Yasuta T, Marschner P, Kandeler E (2006). Response of microbial activity and microbial community composition in soils to long-term arsenic and cadmium exposure. Soil Biol Biochem.

[51] Aronson RB, Thatje S, McClintock JB, Hughes KA (2011). Anthropogenic impacts on marine ecosystems in Antarctica. Ann NY Acad Sci.

[52] Lu Z, Cai M, Wang J, Yang H, He J (2011). Baseline values for metals in soils on Fildes Peninsula, King George Island, Antarctica: the extent of anthropogenic pollution. Environ Monit Assess.

[53] Jerez S, Motas M, Benzal J, Diaz J, Barbosa A (2013). Monitoring trace elements in Antarctic penguin chicks from South Shetland. Mar Pollut Bull.

[54] De Souza MJ, Nair S, Loka Bharathi PA, Chandramohan D (2006). Metal and antibiotic-resistance in psychrotrophic bacterial from Antarctic marine waters. Ecotoxicology.

[55] Arenas FA, Pugin B, Henrı NA, Arenas-salinas MA, Díaz WA, Pérez-Donoso M, Chasteen TG, Muñoz CM, Vásquez CC (2014). Isolation, identification and characterization of highly tellurite-resistant, tellurite-reducing bacteria from Antarctica. Polar Sci.

[56] Pérez-Donoso JM, Monrás JP, Bravo D, Aguirre A, Quest AF, Osorio-Román IO, Aroca RF, Chasteen TG, Vásquez CC (2012). Biomimetic, mild chemical synthesis of CdTe-GSH quantum dots with improved biocompatibility. PLoS One.

[57] Sowers KL, Swartz B, Krauss TD (2013). Chemical mechanisms of semiconductor nanocrystal synthesis. Chem Mater.

[58] Dobias J, Suvorova EI, Bernier-Latmani R (2011). Role of proteins in controlling selenium nanoparticle size. Nanotechnology.

[59] Delalande O, Desvaux H, Godat E, Valleix A, Junot C, Labarre J, Boulard Y (2010). Cadmium—glutathione solution structures provide new insights into heavy metal detoxification. FEBS J.

[60] Dalle-Donne I, Rossi R, Colombo G, Giustarini D, Milzani A (2009). Protein S-glutathionylation: a regulatory device from bacteria to humans. Trends Biochem Sci.

[61] Bai HJ, Zhang ZM, Guo Y, Yang GE (2009). Biosynthesis of cadmium sulfide nanoparticles by photosynthetic bacteria *Rhodopseudomonas palustris*. Colloid Surf B.

[62] Kumar Ram S, Pandian K, Deepak V, Kalishwaralal K, Gurunathan S (2011). Biologically synthesized fluorescent CdS NPs encapsulated by PHB. Enzyme Microb Technol.

[63] Mi C, Wang Y, Zhang J, Huang H, Xu L, Wang S, Fang X, Fang J, Mao C, Xu S (2011). Biosynthesis and characterization of CdS quantum dots in genetically engineered *Escherichia coli*. J Biotechnol.

[64] Manning AJ, Kuehn MJ (2013). Functional advantages conferred by extracellular prokaryotic membrane vesicles. J Mol Microbiol Biotechnol.

[65] Haurat MF, Elhenawy W, Feldman MF (2015). Prokaryotic membrane vesicles: new insights on biogenesis and biological roles. Biol Chem.

[66] Haurat MF, Aduse-Opoku J, Rangarajan M, Dorobantu L, Gray MR, Curtis MA, Feldman MF (2011). Selective sorting of cargo proteins into bacterial membrane vesicles. J Biol Chem.

[67] Pérez-Cruz C, Delgado L, López-Iglesias C, Mercade E (2015). Outer-inner membrane vesicles naturally secreted by gram-negative pathogenic bacteria. PLoS One.

[68] Alipieva E, Zlatov AS, Polischuk VA, Briukhovetskiy AP, Grigoriev DE, Federation R (2013). Influence of quantum dots size dispersion on the fluorescence spectrum. Laser Phys Appl.

[69] Reasoner DJ, Geldreich EE (1985). A new medium for the enumeration and subculture of bacteria from potable water. Appl Environ Microbiol.

[70] Chiong M, Barra R, González E, Vásquez C (1988). Resistance of *Thermus* spp. to potassium tellurite. Appl Environ Microbiol.

[71] Reysenbach AL, Pace NR, Robb FT, Place AR (1995). Reliable amplification of hyperthermophilic archaeal 16S rRNA genes by PCR. Thermophiles.

[72] Suzuki MT, Giovannoni SJ (1996). Bias caused by template annealing in the amplification of mixtures of 16S rRNA genes by PCR. Appl Environ Microb.

[73] Wright ES, Yilmaz LS, Noguera DR (2012). DECIPHER, a search-based approach to chimera identification for 16S rRNA sequences. Appl Environ Microbiol.

[74] Cole JR, Wang Q, Fish JA, Chai B, McGarrell DM, Sun Y, Brown CT, Porras-Alfaro A, Kuske CR, Tiedje JM (2014). Ribosomal Database Project: data and tools for high throughput rRNA analysis. Nucleic Acids Res.

[75] Edgar RC, Drive RM, Valley M (2004). MUSCLE: multiple sequence alignment with high accuracy and high throughput. Nucleic Acids Res.

[76] Tamura K, Stecher G, Peterson D, Filipski A, Kumar S (2013). MEGA6: molecular evolutionary genetics analysis version 6.0. Mol Biol Evol.

[77] Bauer AW, Kirby WM, Sherris JC, Turck M (1966). Antibiotic susceptibility testing by a standardized single disk method. J Clin Pathol.

